# Phenotype- and SSR-Based Estimates of Genetic Variation between and within Two Important *Elymus* Species in Western and Northern China

**DOI:** 10.3390/genes9030147

**Published:** 2018-03-07

**Authors:** Zongyu Zhang, Wengang Xie, Junchao Zhang, Xuhong Zhao, Yongqiang Zhao, Yanrong Wang

**Affiliations:** The State Key Laboratory of Grassland Agro-Ecosystems, Key Laboratory of Grassland Livestock Industry Innovation, Ministry of Agriculture, College of Pastoral Agriculture Science and Technology, Lanzhou University, Lanzhou 730020, China; zhangzy17@lzu.edu.cn (Z.Z.); zhangjch16@lzu.edu.cn (J.Z.); zhaoxh14@lzu.edu.cn (X.Z.); zhaoyq16@lzu.edu.cn (Y.Z.)

**Keywords:** *Elymus*, phenotype, SSR markers, genetic diversity, conservation

## Abstract

*Elymus nutans* and *Elymus sibiricus* are two important perennial forage grasses of the genus *Elymus*, widely distributed in high altitude regions of Western and Northern China, especially on the Qinghai-Tibetan Plateau. Information on phenotypic and genetic diversity is limited, but necessary for *Elymus* germplasm collection, conservation, and utilization. In the present study, the phenotypic and genetic differentiation of 73 accessions of the two species were evaluated using 15 phenotypic traits and 40 expressed sequence tag derived simple sequence repeat markers (EST-SSRs). The results showed that only 7.23% phenotypic differentiation (Pst) existed between the two *Elymus* species based on fifteen quantitative traits. Principal component analysis (PCA) revealed that leaf traits, spike traits, and some seed traits were dominant factors in phenotypic variation. Moreover, 396 (97.8%) and 331 (87.1%) polymorphic bands were generated from 40 EST-SSR primers, suggesting high levels of genetic diversity for the two species. The highest genetic diversity was found in the Northeastern Qinghai-Tibetan Plateau groups. Clustering analysis based on molecular data showed that most accessions of each *Elymus* species tended to group together. Similar results were described by principal coordinates analysis (PCoA) and structure analysis. The molecular variance analysis (AMOVA) revealed that 81.47% and 89.32% variation existed within the geographical groups for the two species, respectively. Pearson’s correlation analyses showed a strong positive correlation between Nei’s genetic diversity and annual mean temperature. These results could facilitate *Elymus* germplasm collection, conservation, and future breeding.

## 1. Introduction

*Elymus* is the largest genus of the tribe *Triticeae* with approximately 150 species widely distributed in temperate regions of the world [1]. Two important species of the genus *Elymus* are hexaploid *Elymus nutans* Griseb with genomes StStHHYY, and tetraploid *Elymus sibiricus* with genomes StStHH. Both are self-pollinating, perennial grasses. Indigenous to North Asia, the two *Elymus* species occur naturally in Eastern Mongolia, Northern Russia, the Himalayas, and parts of Alaska and Canada [2,3]. In China, the two *Elymus* species are widely distributed in the high altitude regions of Western and Northern China, especially in the Qinghai-Tibetan Plateau [2,3,4]. These usually grow in wet meadows, among bushes, along riverbanks, on mountain slopes, and in valleys at altitudes from 1000 up to 4000 m [3,5]. The two *Elymus* species have been widely used as forage crops in cultivated pastures and natural grassland due to their good forage quality, tolerance to diverse environmental conditions as well as important ecological functions in vegetation restoration, soil stabilization, and erosion control [6,7]. Moreover, *Elymus* species are closely related to several important cereal crops such as wheat, barley, and rye, and may serve as potential genetic resource for the improvement of these crops [8,9,10,11].

Despite their ecological and economical importance as potential forage grasses, progress in cultivar development and improvement for the two *Elymus* species has seriously lagged behind crop plants and other forage grasses. In China, only eight *E. sibiricus* cultivars and four *E. nutans* cultivars have been developed from wild materials. The lack of available information about economically valuable traits in the germplasm might be a major reason for the slow progress in cultivar development. The evaluation of phenotypic traits such as plant height, leaf shape, and inflorescence architecture is an initial and essential step for screening germplasms with economically valuable traits, and is also the usual method for determining the relationships among the different accessions [12]. Meanwhile, information about the amount and distribution of genetic diversity is important for germplasm collection in the wild, and for setting up ex situ preserved plant collections of genetic resources that can be used in future plant-breeding programs. Expressed sequence tag derived simple sequence repeat markers (EST-SSRs) are abundant, highly polymorphic, and are easily transferred among related species [13,14,15]. They have been widely used for genetic diversity studies, genetic mapping, and DNA fingerprinting for many crops and forage grasses [7,16,17,18]. 

Previously, several studies have examined the genetic diversity of *E. sibiricus* and *E. nutans* accessions or natural populations. The genetic and geographical divergence of *E. nutans* germplasm from West China were studied using gliadin [19,20] and inter simple sequence repeat (ISSR) markers [2]. The genetic diversity of 52 *E. sibiricus* accessions from the Qinghai-Tibetan Plateau was studied by sequence related amplified polymorphism (SRAP) markers [21]. The genetic variation among and within 24 *E. sibricus* accessions, cultivars, and breeding lines from Northeastern Qinghai-Tibetan Plateau was analyzed by EST-SSR markers [7]. However, most of these previous genetic diversity studies have focused on single *Elymus* species from a small geographical area in the Qinghai-Tibetan Plateau. The extent and patterns of phenotypic and genetic diversity of *E. nutans* and *E. sibiricus* at a large geographical scale are still largely unknown. The Qinghai-Tibetan Plateau is famous for its high elevation, low temperatures, and oxygen concentration where various *Elymus* species are widely distributed. In contrast, the Mongolian Plateau exhibits a dry and windy climate, and also contains a high diversity of *Elymus* species [10]. As a consequence, a comprehensive analysis of the phenotypic and genetic diversity of *Elymus* germplasm, indigenous to the Qinghai-Tibetan Plateau and Mongolian Plateau, may be important not only for the identification and development of *Elymus* germplasm with economically valuable traits, but also for the conservation and utilization of *Elymus* species.

The objectives of present study were to (i) differentiate taxonomically between *E. nutans* and *E. sibiricus*; (ii) estimate the genetic variability of *E. nutans* and *E. sibiricus* geographical groups; and (iii) analyze the genetic structure and relationship of the two *Elymus* species. These results could facilitate *Elymus* germplasm collection, conservation, and future breeding.

## 2. Materials and Methods 

### 2.1. Plant Materials and Phenotype Observation

A total of 37 *E. nutans* accessions and 36 *E. sibiricus* accessions were collected and evaluated based on phenotypic traits and EST-SSR markers. Materials were obtained from the U.S. Department of Agriculture Germplasm Resources Information Network (GRIN), Lanzhou University, Sichuan Agricultural University, and Sichuan Academy of Grassland Science (Supplementary Materials, Table S1). These materials originate from the main distribution areas in the Qinghai-Tibetan Plateau (QTP) and Mongolia Plateau (MP) in China including Tibet, Qinghai, Sichuan, Gansu, Xinjiang, and Inner Mongolia, at altitudes ranging from 500 to 4110 m. (Supplementary Materials, Figure S1). These materials were divided into seven geographical groups based on their origin and ecogeographical environment. These geographical groups were characterized by differences in temperature, precipitation, altitude, and elevation. *E. nutans* accessions were divided into four geographical groups including of Northeastern Qinghai-Tibetan Plateau (NEQ1), Northwestern Qinghai-Tibetan Plateau (NWQ1), Southeastern Qinghai-Tibetan Plateau (SEQ1) and Mongolian Plateau populations (MP1). *E. sibiricus* accessions were divided into three geographical groups: Northeastern Qinghai-Tibetan Plateau (NEQ2), Southeastern Qinghai-Tibetan Plateau (SEQ2), and Mongolian Plateau (MP2).

Approximately 30 seeds of each accession were germinated and seedlings were maintained in a greenhouse under a 25/15 °C day/night temperature regimes until they were eight weeks old. Twenty individual plants of each accession were transplanted to the Yuzhong experimental field of Lanzhou University, Gansu, China (latitude 35°34′ N, longitude 103°34′ E, elevation 1720 m). After transplanting, plants were watered immediately, and no fertilizer was applied after transplanting. 

In the second year, a total of 730 individual plants (10 individuals of each accession) of the two species were selected for evaluating 15 phenotypic traits. Flag leaf length (FL), flag leaf width (FW), length of second leaf from bottom (LB), width of second leaf from bottom (WB), plant height (PH), culm node number (CN), tiller number (TN), culm diameter (CD) spike length (SL), floret number per spike (FN), length of lemma (LL), width of lemma (WL), awn length of lemma (AL), 1000-seed weight (SW1) were measured using the methods described by Zhao et al. [22]. Seed shattering (SS) was determined by measuring pedicel breaking tensile strength (BTS) required to detach the seeds from the pedicels. Thirty randomly chosen spikelets of each plant were examined at 4 weeks after heading, and their average BTS values were calculated.

### 2.2. DNA Extraction and Polymerase Chain Reaction Amplification

Ten individuals of each accession were sampled and pooled together for DNA extraction using the SDS (sodium dodecyl sulfate) method [23]. The quantity and quality of DNA samples was determined using the NanoDrop ND1000 spectrophotometer (Thermo Scientific, Waltham, MA, USA) and agarose gel electrophoresis, then diluted to 25 ng/µL and stored at −20 °C. A total of 40 EST-SSR primers were used in the study including 25 primers selected from the *E. lanceolatus* (Elw hereafter), *Pseudoroegneria* (Ps hereafter), and *Leymus* (Lt hereafter), and 15 newly developed *E. sibiricus* (ES hereafter) EST-SSR primers reported by Zhou et al. [15]. The twenty-five Elw, Ps and Lt markers were developed by Bushman et al. [24]. The PCR amplification and SSR genotyping as well as the methods of electrophoresis process were carried out as described by Zhou et al. [15] and Xie et al. [7].

### 2.3. Data Analysis

The mean value, standard deviation, coefficient of variation (CV), Shannon’s diversity index (H’), and principal component analysis (PCA) of phenotypic traits were analyzed using Excel 2013 (Microsoft Inc., Redmond, WA, USA) and SPSS 19.0 software (SPSS Inc., Chicago, IL, USA). Shannon’s diversity index was calculated as follows: H’ = –∑ *pi* Ln *pi*, where *pi* is the proportion of each phenotypic trait [25,26]. According to the mean value of each trait for each accession, a heatmap was constructed using the Heatmap Illustrator (HemI 1.0) program (Beijing Institude of Genomics, CAS, Beijing, China) [27]. All phenotypic data were normalized under the logarithmic relations (log 2) first due to the different conditions of measurement, then they were used to map the visualized color matrix (73 rows × 15 columns). The color scale ranged from 7.23 to 0.02 (red to blue). Each line and column represented different accessions and different traits, respectively. Hierarchical clustering was performed based on the average linkage method and the similarity metric with Pearson distance. In addition, a Pearson correlation analysis among the different traits was implemented using SPSS 19.0 software. The phenotypic differentiation coefficient (Pst) was calculated as follows: Pst = (σ^2^_t/s_)/(σ^2^_t/s_ + σ^2^_s_), where σ^2^_t/s_ is the variance portion among populations and σ^2^_s_ is the variance portion within populations [28]. 

The amplified bands were considered as present (1) and absent (0), and the binary matrix data produced in an Excel file was used for further statistical analysis. Polymorphism information content (PIC) were calculated as follows: PIC = 1 − *p*^2^ − *q*^2^, where *p* is the frequency of present band and *q* is the frequency absent band [29]. Genetic diversity parameters including observed number of alleles (Na), effective number of alleles (Ne), Shannon’s information index (I), and Nei’s genetic diversity (H) among seven populations were calculated by using GenAlEx 6.5 (Canberra Australia) [30]. The pairwise genetic differentiation and genetic distance among these populations were analyzed using the POPGENE 1.31 program (Edmonton, AB, Canada) [31]. The binary data was analyzed using qualitative routine to generate Jaccard’s genetic similarity coefficient (GS). Based on the GS matrix, a principal coordinates analysis (PCoA) was constructed using DCENTER module in NTSYS (version 2.10), and a dendrogram was constructed using the unweighted pair group method with arithmetic mean (UPGMA) of NTSYS [32]. A bootstrap analysis with 1000 replicates was performed to obtain the confidence of branches of the cluster tree using the Winboot software [33]. The genetic structure of the 73 *Elymus* accessions was analyzed using STRUCTURE v 2.3.4 software (Stanford, CA, USA) with the ‘admixture mode’, burn-in period of 10,000 interactions and a run of 100,000 replications of Markov chain Monte Carlo (MCMC) after burn in [34]. For each run, 20 independent runs of STRUCTURE were performed with the number of clusters (K) varying from 1 to 11. Mean L (K) and delta K (ΔK) were estimated using the method described by Evanno et al. [35]. Maximum likelihood and delta K (ΔK) were used to determine the optimum number of groups. AMOVA software (version 1.55) (Geneva, Switzerland)was employed to analyze the distribution of genetic variance within and among geographic groups [36]. The Shannon differentiation coefficient (Gst) was calculated according to the following formula: Gst = (Hsp − Hpop)/Hsp (Hsp, total Shannon information index; Hpop, average Shannon information index within the population). Gene flow (Nm) was calculated as Fst (Nm = (1 − Fst)/4Fst) [37]. In addition, to determine the relationships between genetic diversity and environmental factors (annual mean temperature, annual precipitation, altitude, and latitude), a linear regression analysis was conducted on SPSS 19.0 (SPSS Inc., Chicago, IL, USA). GenAlEx6.5 (Canberra, Australia) was used to analyze the correlation between genetic distance and geographic distances by a Mantel test [38]. 

## 3. Results

### 3.1. Phenotypic Differentiation between Two Elymus Species

The phenotypic variations of 73 *Elymus* accessions were studied using 15 traits. Based on the phenotypic differentiation coefficient (Pst) analysis, 12.67% and 17.10% phenotypic variation existed among *E. nutans* and *E. sibiricus* groups, respectively (Table 1). The greatest phenotypic differentiation was found for seed shattering (Pst = 23.43%), followed by awn length (Pst = 18.44%), culm diameter (Pst = 16.56%), and tiller number (Pst = 11.72%) when all the 15 traits between the two species were compared. Many traits showed a considerable level of variation for two *Elymus* species (Table 2). In *E. nutans*, shannon’s diversity index (H’) ranged from 1.64 to 2.23, with an average of 2.03. Four traits: flag leaf length (FL), tiller number (TN), floret number per spike (FN), and seed shattering (SS) had a coefficient of variation (CV) greater than 15%. The greatest phenotypic variation was found for seed shattering (CV = 28.11%). For *E. sibiricus*, the H’ value varied from 1.51 to 2.26, with an average of 2.06. The greatest phenotypic variation was found for tiller number (CV = 19.97%), followed by floret number per spike (CV = 15.47%) and seed shattering (CV = 15.45%). Length of lemma (LL) showed the lowest phenotypic variation in *E. nutans* (CV = 4.99%) and *E. sibiricus* (CV = 7.12%), respectively. 

**Figure 1:**
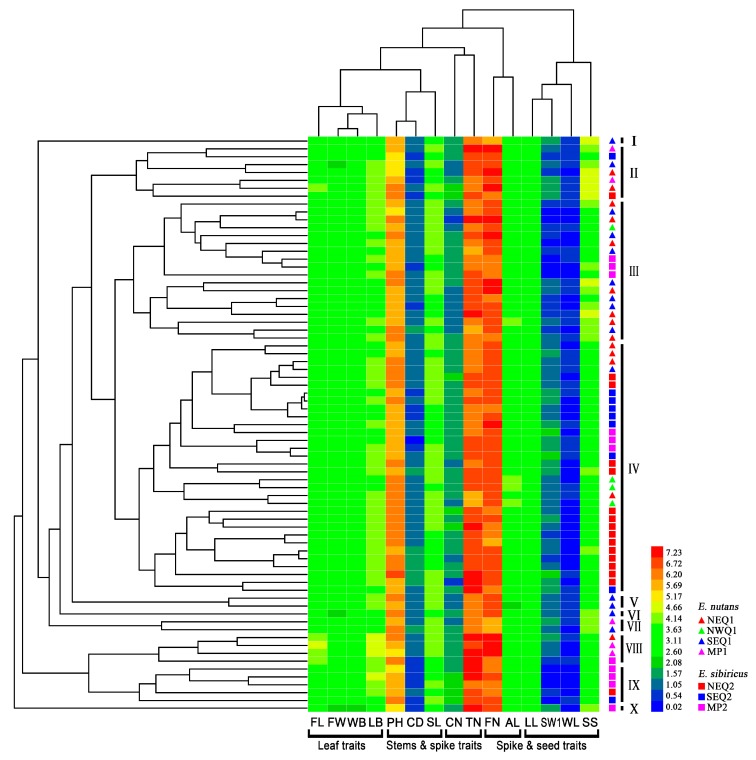
Clustering and Heatmap analysis of the 73 *Elymus* accessions using fifteen phenotypic traits. All of the 73 *Elymus* accessions were clustered in ten subgroups. Each line and column represents different accessions and different traits, respectively.

Clustering with 15 traits formed two major groups; Group 1 was divided into four subgroups: leaf traits group (FL, FW, WB, and LB); stem and spike traits group (PH, CD, and PL); stem traits group (CN and TN); and spike and seed traits group (FN and AL). The remaining four seed traits (LL, SW1, WL, and SS) were assigned to another group (Figure 1). Clustering analysis showed that 73 accessions were assigned to ten subgroups. For *E. nutans* accessions, within each cluster there were no obvious correlations between the phenotypic traits and geographic origins. Some *E. sibiricus* accessions from eastern groups of the Qinghai-Tibetan Plateau (NEQ2 and SEQ2) had similar phenotypic performance (Cluster IV). In *E. nutans*, the principal component analysis (PCA) showed that the first three principal components could explain 60.77% of the total phenotypic variation. PC1 was mainly involved in leaf traits, PC2 was mainly involved in seed traits (Table 2). In contrast, for *E. sibiricus*, three principal components explained 54.24% of the total phenotypic variation. PC1 was focused on leaf traits, and PC2 on spike and seed traits. The correlation coefficient analysis showed significantly positive correlations among the four *Elymus* leaf traits: Flag leaf length (FL), flag leaf width (FW), length of second leaf from bottom (LB), width of second leaf from bottom (WB), while seed shattering showed a negative correlation with most traits detected (Supplementary Materials, Table S2). 

### 3.2. Genetic Diversity and Genetic Relationship between Two Elymus Species

In this study, forty EST-SSR primers generated 405 and 380 bands for *E. nutans* and *E. sibiricus*, respectively (Supplementary Materials, Figure S2). The polymorphic information content (PIC) ranged from 0.16 to 0.45, with an average of 0.32 for *E. nutans*, and the PIC value varied from 0.03 to 0.43, with an average of 0.23 for *E. sibiricus* (Supplementary Materials, Table S3). In general, *E. nutans* had a relatively higher level of genetic diversity (PPL = 68.15%, Na = 1.49, Ne = 1.36, I = 0.33, H = 0.22) than *E. sibiricus* at species level (Table 3)*.* Genetic variation was found between *E. nutans* and *E. sibiricus* in the same geographic region. *E. nutans* had a higher level of genetic diversity in Northeastern Qinghai-Tibetan Plateau (NEQ) (PPL = 88.64%), Southeastern Qinghai-Tibetan Plateau (75.56%), and Mongolian Plateau (MP) (PPL = 69.88%) groups. The highest genetic diversity was found in Northeastern Qinghai-Tibetan Plateau groups (NEQ) for both the *Elymus* species (PPL = 88.64% and 81.84%, respectively). Correspondingly, the lowest genetic diversity was found in northwestern Qinghai-Tibetan Plateau (NWQ1) *E. nutans* groups (PPL = 38.52%) and Southeastern Qinghai-Tibetan Plateau (SEQ2) *E. sibiricus* groups (PPL = 53.68%).

**Figure 2:**
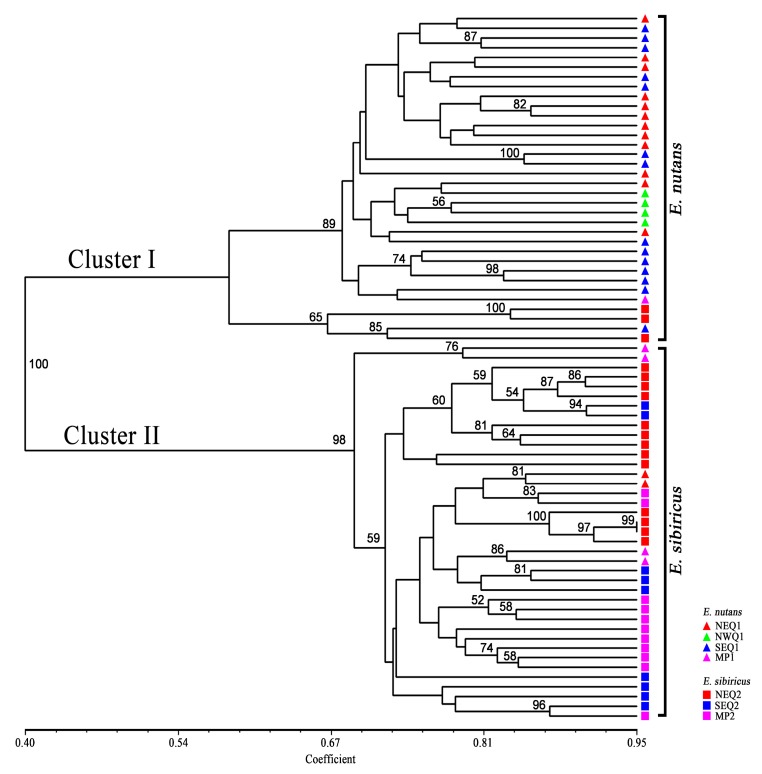
Unweighted pair group method with arithmetic mean (UPGMA)-derived dendrogram of 73 *Elymus* accessions based on Jaccard’s genetic similarity, only bootstrap values higher than 50% are presented. The species and geographical groups each individual belongs to refer to right codes.

**Figure 3:**
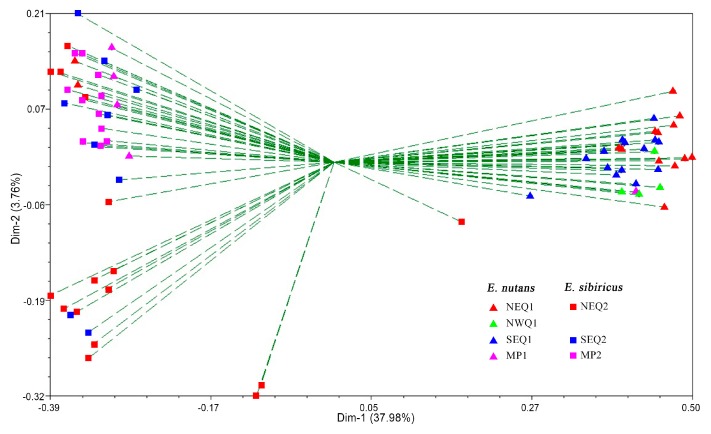
Principal coordinates analysis (PCoA) for the first and second coordinates estimated for EST-SSR markers using Jaccard’s genetic similarity matrix for seven groups of *Elymus* accessions. Different groups were distinguished by different colors and shapes. Red triangle: NEQ1; green triangle: NWQ1; blue triangle: SEQ1; pink triangle: MP1; red square: NEQ2; blue square: SEQ2; pink square: MP2.

The unweighted pair-group method using arithmetic averages (UPGMA) cluster analysis showed that all accessions were clustered into two major clusters with a bootstrapping value of 100% (Figure 2). Cluster I contained a total of 31 *E. nutans* accessions and 3 *E. sibiricus* accessions. Thirty-three *E. sibiricus* accessions and 6 *E. nutans* accessions were assigned to Cluster II. In general, the vast majority of accessions (83.8% *E. nutans* accessions and 91.7% *E. sibiricus* accessions) could easily be distinguished using selected EST-SSR markers. The results observed in the principal coordinate analysis (PCoA) were in agreement with the UPGMA analysis. The first three principle components explained 44.98% of the total variation. A moderate, but clear separation between *E. nutans* and *E. sibiricus* was revealed (Figure 3). Two *E. nutans* accessions from Northeastern Qinghai-Tibetan Plateau and four from Mongolian Plateau were grouped with *E. sibiricus*.

### 3.3. Genetic Structure between and within the Two Elymus Species

**Figure 4:**
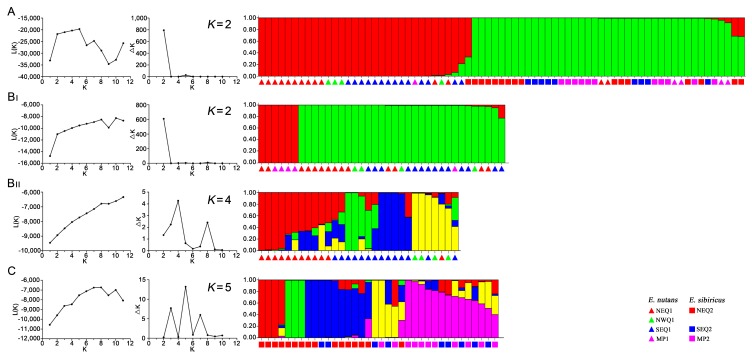
Population structure of 73 *Elymus* accessions based on Bayesian inferred from STRUCTURE program with 40 EST-SSR markers data set. Each structure was described by using two graphics of the process to detect the optimum *K* value. One was the mean L (*K*) over 20 runs for each *K* value (1–11), another was the maximum delta *K* values used to determine the uppermost level of structure for *K* ranging from 2 to 10. Different code and corresponding vertical lines represent an individual genotype and different colors represent genetic stock. (**A**) 73 accessions were clustered into two groups at *K* = 2. Red zone: 31 *E. nutans* accessions and 1 *E. sibiricus* accessions; Green zone: 35 *E. sibiricus* accessions and 6 *E. nutans* accessions; (**B_I_**) *E. nutans* accessions were clustered into two groups (*K* = 2); (**B_II_**) *E. nutans* accessions from QTP were clustered into four groups (*K* = 4); and (**C**) *E. sibiricus* accessions were clustered into five groups (*K* = 5).

The genetic structure of the 73 *Elymus* accessions was analyzed using STRUCTURE software. Based on maximum likelihood and delta K (ΔK) values, the optimal number of groups was two (Figure 4). As shown in Figure 4A, all 73 *Elymus* accessions were divided into two subpopulations, corresponding to *E. nutans* and *E. sibiricus* subgroups with a few admixed lines, which was consistent with clustering based on genetic distance. We further investigated the internal genetic structure of *E. nutans* and *E. sibiricus*, respectively (Figure 4B,C). Thirty-seven *E. nutans* accessions were assigned to two major groups when K was two (Figure 4B_І_). Group 1 consisted of six accessions, mainly from the Mongolia Plateau; Group 2 consisted of 31 accessions predominantly from the Qinghai-Tibetan Plateau. Thirty *E. nutans* accessions from the Qinghai-Tibetan Plateau could be clearly divided into four subgroups when *K* was 4 (Figure 4B_II_). Eleven *E. nutans* accessions from Northeastern Qinghai-Tibetan Plateau (ENQ1) and two accessions from Southeastern Qinghai-Tibetan Plateau (SEQ1) were assigned to Cluster 1. Nine accessions (69.2%) in this group were categorized as having admixed ancestry. Ten SEQ1 accessions were assigned to Clusters 2 and 3, the remaining seven accessions were assigned to Cluster 4 based on the proportional membership (>0.5). There was no obvious relationship between geographic location and genetic structure in *E. sibiricus* accessions (Figure 4C), as demonstrated by 15 accessions from the Northeastern Qinghai-Tibetan Plateau where four in Cluster 1, three in Cluster 2, and eight in Cluster 3. Cluster 3 also included two accessions from the Southeastern Qinghai-Tibetan Plateau. Most admixed lines (42%) from the Mongolian Plateau (MP) and Southeastern Qinghai-Tibetan Plateau (SEQ) were assigned to Clusters 4 and 5.

### 3.4. Genetic Variation among and within Geographic Groups

Genetic differentiation (Gst) and genetic distance among the four *E. nutans* and three *E. sibiricus* geographical groups were calculated based on Nei’s original measures (Table 4). Among the *E. nutans* groups, the greatest Gst value (0.37) and genetic distance (0.31) were found between Northwestern Qinghai-Tibetan Plateau (NWQ1) and the Mongolian Plateau (MP1). Among the *E. sibiricus* groups, the greatest genetic differentiation (Gst = 0.09) was found between Southeastern Qinghai-Tibetan Plateau (SEQ2) and the Mongolian Plateau (MP2). 

Analysis of molecular variance (AMOVA) was conducted to further evaluate the partitioning of genetic differentiation among and within the geographic groups. The results showed that less than 20% of the variation occurred among the geographic groups, whereas more than 80% of total variance existed within geographic groups (81.47% for *E. nutans*, 89.32% for *E. sibiricus*) (Table 5). Moreover, the average Shannon differentiation coefficient was 0.33 for the *E. nutans* geographic group and 0.25 for *E. sibiricus* group with a higher gene flow (Nm = 1.96) across all *E. sibiricus* geographic groups.

### 3.5. Association between Habitat Parameters and Genetic Variation

**Figure 5:**
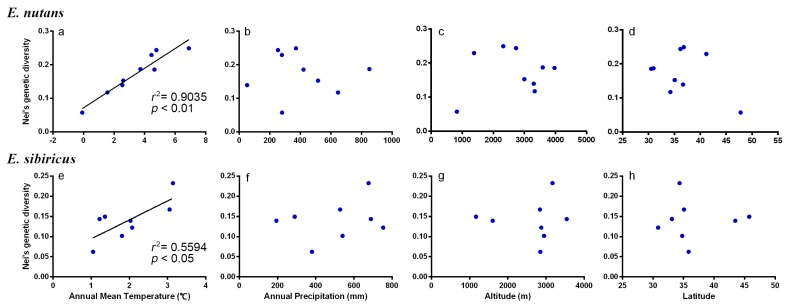
Regression analysis between Nei’s genetic diversity (H) and environmental factors for *E. nutans* and *E. sibiricus*. A regression line was fitted only for analyses with *p* < 0.05.

Pearson’s correlation analyses showed that a strong positive correlation between Nei’s genetic diversity and annual mean temperature (*r* = 0.95 for *E. nutans*, *p* < 0.01; *r* = 0.78 for *E. sibiricus*, *p* < 0.05) (Table 6 and Figure 5). While a weakly positive correlation was found between genetic diversity and annual precipitation, altitude as well as latitude change for *Elymus* groups.

## 4. Discussion

### 4.1. Phenotypic and Genetic Diversity between the Two Elymus Species 

The measurement of phenotypic traits is considered as an important and fundamental method for diversity analysis [13,39]. Our results showed a relatively high level of phenotypic diversity among the 73 *Elymus* accessions. Similar results were also found in previous studies. Chen et al. evaluated the phenotypic diversity of 54 *E. nutans* accessions from Qinghai-Tibetan Plateau based on thirty phenotypic traits, and found 16.05% phenotypic variation [40]. Among these phenotypic traits, leaf traits, spike traits, and some seed traits were dominant factors of phenotypic difference in *Elymus* species [16,22,40,41]. These traits might be useful for the description and determination of the relationship among different *Elymus* species. However, the process of phenotypic assessment is time-consuming, and the effectiveness and accuracy of assessment is limited due to the influence of environment factors. Compared with phenotypic assessment, molecular marker technology is an efficient supplement and alternative to phenotypic measurements. Our results showed relatively higher level of genetic diversity of *E. sibiricus* geographical groups (average PPL = 63.16%) when compared with a previous study. Zhang et al. employed sixteen start codon targeted (SCoT) markers for assessing the genetic diversity of *E. sibiricus*, and found 37.63% genetic variation among the five geographical groups [42]. Chen et al. analyzed the genetic diversity of 50 *E. nutans* accessions from the eastern Qinghai-Tibetan Plateau using simple sequence repeat markers (SSRs), and found 91.38% genetic diversity [43]. In this study, although only 14 *E. nutans* accessions from Southeastern Qinghai-Tibetan Plateau were analyzed, more than 75% genetic diversity was found. Therefore, the results of the present study showed that the two *Elymus* species had high level of genetic diversity, and also indicated that SSR markers were efficient in assessing the genetic diversity of *E. nutans* and *E. sibiricus* accessions. 

### 4.2. Genetic Variation of Geographic Groups and Its Correlation to Environment Factors

In general, *E. nutans* had a relatively higher genetic diversity when compared with *E. sibiricus*, possibly due to the additional Y genome in the hexaploid *E. nutans* and a wider geographic range. Previous studies showed that genetic variability is usually higher in polyploidy species [44]. Based on the AMOVA results, a high level of genetic variation (>80%) was found within the geographical groups of the two *Elymus* species. Similar results were also found in other self-pollinating *Elymus* species such as *E. trachycaulus* and *E. glaucus*. Steven et al. analyzed four *E. trachycaulus* populations and found 85% within-population variation using SSR markers [45]. Wilson et al. revealed that 60% of the total genetic variation existed within populations in *E. glaucus* [46]. The distribution of genetic differences and the patterns of genetic diversity among and within plant populations can be directly and/or indirectly influenced by some factors: plant reproductive mode, random gene flow, gene drift among populations as well as sample size [3,7]. Previous studies have found that seed dispersal of *Elymus* species played a more important role than pollen movement. Seed dispersal happens more commonly between populations with the same regions at similar altitudes [47]. There are a number of ways in which long-distance seed dispersal can occur including wind-mediated, animal-mediated, and water-mediated seed dispersal. Given the fact that the two species are self-pollinating plant species, we inferred that a significant amount of seed-mediated gene flow among *Elymus* populations has contributed to their population genetic structure. In addition, the sample size used for the genetic diversity study may have also had a significant effect on the measurements of genetic variability among and within populations [7]. A small sample size from some geographic groups (e.g., four accessions from the Northwestern Qinghai-Tibetan Plateau) may have contributed to a lower estimate of genetic diversity. 

Meanwhile, environment parameters are highly correlated with the magnitude and distribution of genetic diversity [7,43]. A species must accumulate more genetic diversity in order to adapt itself to diverse environmental pressures [37]. In this study, a correlation between genetic diversity and the annual mean temperature, annual precipitation, altitude, and latitude change was found for *Elymus* accessions. In the Qinghai-Tibet Plateau, towering mountains and deep valleys could also serve as genetic barriers of pollinator movement and seed dispersal, which could result in genetic difference among accessions especially in large geographical regions. Therefore, the diversity of geographical origin or ecological conditions of *Elymus* accessions in this study could explain the relatively high level of genetic diversity. 

### 4.3. Conservation Implications

Phenotypically and genetically diverse germplasm is a potentially valuable source for the improvement of desired agronomic trait such as seed yield, quality and stress tolerance. To broaden the genetic base, one of the important strategies is to screen and develop novel germplasm or breeding lines with desired traits. Wild germplasm could provide advantageous alleles like improved disease resistance, stress resistance, and higher yield for modifying crops by hybridization and introgression [48]. Past studies in wheatgrass [49] and orchard grass [50] have demonstrated incorporation of useful genetic and phenotypic diversity into cultivated material. In addition, Zhang et al. used two wild *E. sibiricus* genotypes with genetic and phenotypic differences to generate F_1_ hybrids. The molecular and phenotypic diversity analysis of F_1_ population revealed rich genetic and phenotypic variations. Seed shattering, flag leaf length and width showed the positive heterosis [51]. Zhao et al. created five hybrid populations by crossing seven wild *E. sibiricus* genotypes with seed shattering variation, and found high level of genetic diversity in hybrid populations using SSR markers, and the results also suggested more genetic variation could be captured in hybrid populations by crossing breeding [52]. A previous report showed genetic distance between germplasm can be a predictor of combining ability, and suggested that genetically differentiated pool of germplasm was important for plant genetic improvement [50]. Based on our data, *Elymus* accessions from the Northwestern groups of the Qinghai-Tibetan Plateau had highest level of genetic diversity. The greatest phenotypic differentiation was found for seed shattering. Therefore, these wild accessions with affluent genetic diversity and desired traits could be used as important genetic resources for future *Elymus* breeding programs.

## 5. Conclusions

This study showed a high level of phenotypic and genetic diversity of 73 *Elymus* accessions in Western and Northern China. *E. nutans* had relatively higher genetic diversity than *E. sibiricus* at the species level. The ecological factors such as annual temperature, precipitation, altitude played a role in the divergence. More genetic variation of the two *Elymus* species can be captured when sampling a larger number of germplasm from the Northeastern Qinghai-Tibetan Plateau. Meanwhile, these traits related to biomass yield and seed yield should be considered in future breeding programs.

## Figures and Tables

**Table 1:** Variance portion of each trait among geographic groups (AP) and within geographic groups (WP), phenotypic differentiation coefficient (Pst) for 15 traits within and between two *Elymus* species (Pst (S)).

Traits	*E. nutans*					*E. sibiricus*					Pst (S)
	Variance Portion		Percent of Variance Portion (%)		Pst	Variance Portion		Percent of Variance Portion (%)		Pst	
	AP (σ^2^_t/s_)	WP (σ^2^_s_)	AP (σ^2^_t/s_)	WP (σ^2^_s_)		AP (σ^2^_t/s_)	WP (σ^2^_s_)	AP (σ^2^_t/s_)	WP (σ^2^_s_)		
FL	6.23	14.43	21.34	49.40	30.17%	2.14	8.67	10.99	44.53	19.80%	5.02%
FW	1.19	2.94	18.52	45.93	28.74%	0.01	0.05	15.41	60.29	20.37%	1.86%
LB	3.46	15.08	11.77	51.34	18.65%	2.09	5.92	11.91	33.71	26.10%	6.60%
WB	0.88	2.84	14.83	47.59	23.76%	0.02	0.04	23.20	49.94	31.73%	3.93%
PH	29.18	138.09	11.57	54.78	17.44%	210.93	155.07	39.67	29.16	57.63%	0.00%
CN	0.02	0.34	3.59	53.45	6.29%	0.02	0.48	0.00	55.21	0.00%	16.56%
TN	22.41	1175.19	0.91	47.91	1.87%	81.55	823.07	3.49	35.24	9.02%	11.72%
CD	0.00	0.07	0.00	29.55	0.00%	0.35	0.23	0.00	0.25	0.00%	8.94%
SL	0.65	5.69	0.00	48.67	0.00%	0.69	7.88	5.13	58.23	8.09%	5.10%
FN	128.25	797.29	7.04	43.75	13.86%	170.48	432.22	15.25	38.67	28.29%	0.00%
LL	0.01	0.01	10.95	46.86	18.95%	0.01	0.01	18.78	35.90	34.35%	3.79%
WL	0.01	0.01	0.21	47.04	0.44%	0.00	0.00	0.00	0.91	0.00%	0.00%
AL	0.01	0.08	11.60	65.66	15.02%	0.01	0.03	3.53	60.37	5.53%	18.44%
SW1	0.04	0.42	6.78	77.78	8.02%	0.06	1.17	4.61	87.56	5.00%	3.10%
SS	1.50	20.64	1.76	24.15	6.78%	1.08	9.09	5.09	43.14	10.57%	23.43%
Mean	12.92	144.87	8.06	48.92	12.67%	31.29	96.26	10.47	42.21	17.10%	7.23%

**Table 2:** The phenotypic diversity coefficient of 15 traits and the first three principal components of phenotypic variation for *E. nutans* (left) and *E. sibiricus* (right) accessions.

Traits		Range		Mean Value		Standard Deviation		CV		H’		PCA of *E. nutans*			PCA of *E. sibiricus*		
		EN	ES	EN	ES	EN	ES	EN	ES	EN	ES	PC1	PC2	PC3	PC1	PC2	PC3
Leaf traits	FL (cm)	7.20–25.60	7.50–23.40	12.38	12.29	1.93	1.64	15.66%	13.61%	2.15	2.08	0.78	−0.37	0.32	0.71	−0.42	−0.11
	FW (mm)	4.90–13.80	5.30–12.90	9.31	9.12	1.01	0.99	11.28%	11.14%	2.11	2.17	0.86	0.15	−0.17	0.88	−0.26	0.20
	LB (cm)	12.60–30.80	13.30–25.60	19.00	19.03	2.18	1.63	11.62%	8.54%	1.90	2.08	0.78	−0.41	0.19	0.81	−0.29	−0.11
	WB (mm)	6.10–15.10	5.90–13.80	10.91	10.44	1.04	1.04	9.88%	10.26%	2.23	2.07	0.84	0.09	−0.33	0.82	−0.18	0.30
Stems traits	PH (cm)	44.30–99.60	46.70–114.90	71.09	72.12	6.56	7.55	9.54%	10.70%	1.98	2.08	0.14	0.66	−0.49	0.50	0.34	−0.33
	CN (No.)	1.00–4.50	1.00–5.30	2.97	3.75	0.39	0.49	13.25%	13.69%	2.07	2.22	0.60	0.27	0.21	0.18	−0.34	0.43
	TN (No.)	65.60–214.5	79.30–198.60	110.95	130.76	23.10	25.92	20.46%	19.97%	1.93	2.09	0.36	−0.49	0.15	0.15	−0.11	−0.36
	CD (mm)	1.60–3.10	1.40–3.40	2.43	2.30	0.29	0.29	11.89%	12.33%	1.87	1.98	0.26	0.25	−0.65	0.57	0.46	−0.51
Spike and seed traits	SL (cm)	12.30–24.70	11.10–24.70	18.63	17.57	1.57	1.46	8.52%	8.47%	2.10	2.21	0.55	−0.03	−0.13	0.33	0.69	−0.04
	FN (No.)	56.10–211.3	68.20–155.00	128.57	113.55	24.17	17.79	19.88%	15.47%	2.11	2.12	0.48	−0.54	0.17	0.12	0.30	0.50
	LL (mm)	7.30–10.60	7.30–12.30	9.21	9.59	0.46	0.69	4.99%	7.12%	2.15	2.05	0.35	0.68	0.26	0.25	0.66	−0.15
	WL (mm)	1.00–1.80	1.10–1.70	1.49	1.41	0.14	0.13	9.55%	9.43%	2.22	1.90	0.17	0.64	0.54	0.09	−0.05	0.69
	AL (mm)	5.00–19.00	8.90–15.60	13.45	11.52	1.14	1.02	8.67%	9.03%	2.13	2.26	0.20	0.19	−0.45	0.15	0.26	0.49
	SW1 (g)	1.10–4.00	1.00–5.00	2.36	2.71	0.19	0.21	8.51%	8.64%	1.81	1.51	0.37	0.65	0.47	0.12	0.71	0.30
	SS (gf)	11.70–33.00	9.30–28.20	19.54	14.74	5.52	2.21	28.11%	15.45%	1.64	2.07	−0.43	0.21	0.48	0.02	0.51	0.29
	Mean							12.79%	11.59%	2.03	2.06						
	EV											4.27	2.80	2.05	3.47	2.67	2.00
	%V											28.44	18.64	13.70	23.13	17.77	13.34
	C%											28.44	47.08	60.77	23.13	40.90	54.24

EN, *E. nutans* accessions; ES, *E. sibiricus* accessions; FL, flag leaf length; FW, flag leaf width; LB, length of second leaf from bottom; WB, width of second leaf from bottom; PH, plant height; CN, culm node number; TN, tiller number; CD, culm diameter; SL, spike length; FN, floret number per spike; LL, length of lemma; WL, width of lemma; AL, awn length of lemma; SW1, 1000 seed weight; SS, seed shattering; CV, coefficient of variation; H’, Shannon’s diversity index; EV, eigenvalues; %V, % of variance; C%, Cumulative %.

**Table 3:** Environmental parameters of geographic regions and genetic variability within four *E. nutans* and three *E. sibiricus* groups.

Species	Pop	AMT (°C)	AP (mm)	A (m)	L (N)	N	NPL	PPL (%)	NPB	Na	Ne	I	H
*E. nutans*	NEQ1	4.00	446.20	2819.30	35°63′	14	359	88.64	4	1.82 ± 0.03	1.45 ± 0.02	0.42 ± 0.01	0.27 ± 0.01
	NWQ1	2.50	51.30	3300.00	36°67′	4	156	38.52	0	1.02 ± 0.04	1.24 ± 0.02	0.21 ± 0.01	0.14 ± 0.01
	SEQ1	4.20	636.50	3779.30	30°75′	14	306	75.56	5	1.63 ± 0.03	1.36 ± 0.02	0.34 ± 0.01	0.22 ± 0.01
	MP1	2.60	279.70	1158.00	43°82′	5	283	69.88	11	1.50 ± 0.04	1.38 ± 0.02	0.35 ± 0.01	0.23 ± 0.01
	Mean						276.0	68.15	5.0	1.49 ± 0.02	1.36 ± 0.01	0.33 ± 0.01	0.22 ± 0.01
									47				
*E. sibiricus*	NEQ2	2.30	521.80	2930.90	35°06′	16	311	81.84	62	1.78 ± 0.03	1.37 ± 0.02	0.36 ± 0.01	0.23 ± 0.01
	SEQ2	1.60	717.30	3244.40	32°10′	9	204	53.68	3	1.29 ± 0.04	1.29 ± 0.02	0.27 ± 0.01	0.18 ± 0.01
	MP2	1.70	237.60	1405.80	44°52′	11	205	53.95	5	1.31 ± 0.04	1.28 ± 0.02	0.26 ± 0.01	0.17 ± 0.01
	Mean						240.0	63.16	23.30	1.46 ± 0.02	1.31 ± 0.01	0.29 ± 0.01	0.19 ± 0.01
									40				

AMT, annual mean temperature; AP, annual precipitation; A, altitude; L, latitude; N, sample size; NPL, the number of polymorphic loci; PPL, the percentage of polymorphic loci; NPB, number of private bands; Na, observed number of alleles; Ne, effective number of alleles; I, Shannon’s Information index; H, Nei’s genetic diversity.

**Table 4:** Pairwise genetic differentiation (Gst) (below diagonal) and pairwise genetic distance (above diagonal) among four *E. nutans* and three *E. sibiricus* groups based on Nei’s original measures.

***E. nutans***	**NEQ1**	**NWQ1**	**SEQ1**	**MP1**
NEQ1		0.11	0.05	0.14
NWQ1	0.17		0.09	0.31
SEQ1	0.07	0.17		0.23
MP1	0.17	0.37	0.26	
***E. sibiricus***	**NEQ2**	**SEQ2**	**MP2**	
NEQ2		0.04	0.05	
SEQ2	0.08		0.04	
MP2	0.09	0.09		

**Table 5:** Analysis of molecular variance (AMOVA) of in four *E. nutans* and three *E. sibiricus* geographic groups.

Species	Source of Variance	Degrees of Freedom	Sum of Squares	Mean Square	Variance Components	Total Variance	*p*-Value
*E. nutans*	Among geographic groups	3	503.15	167.72	13.07	18.53%	<0.001
	Within geographic groups	33	1897.06	57.49	56.49	81.47%	<0.001
	Total	36	2400.21		69.56		
*E. sibiricus*	Among geographic groups	2	218.15	109.08	5.45	10.68%	<0.001
	Within geographic groups	33	1505.43	45.62	45.62	89.32%	<0.001
	Total	35	1723.58		51.07		

**Table 6:** Regression analysis between Nei’s genetic diversity (H) and environmental factors.

Species	Pearson Coefficient	Annual Mean Temperature	Annual Precipitation	Altitude	Latitude
*E. nutans*	*r*	0.95	−0.03	0.16	−0.39
	*p*	<0.01 **	0.95 ^ns^	0.68 ^ns^	0.29 ^ns^
*E. sibiricus*	*r*	0.78	0.28	0.04	0.03
	*p*	0.03 *	0.51 ^ns^	0.92 ^ns^	0.95 ^ns^

** *p* < 0.01, * *p* < 0.05, ^ns^ = not significant.

## Supplementary Materials

The following are available online at http://www.mdpi.com/2073-4425/9/3/147/s1. Figure S1: The geographical distribution of *E. nutans* and *E. sibiricus* accessions used in the study, Figure S2: The amplification results of 73 *Elymus* accesions by Elw2807s159, Table S1: Details of seventy-three *Elymus* accessions used in the study, Table S2: Correlation coefficients obtained from pairwise comparisons of 15 phenotypic traits of *E. nutans* (above diagonal) and *E. sibiricus* (below diagonal), Table S3: EST-SSR markers used in this study, amplification results, assigned linkage groups of *Elymus.*
