# Supplementary material for: Phenotype- and SSR-Based Estimates of Genetic Variation between and within Two Important Elymus Species in Western and Northern China
Source: Genes (Basel). 2018 Mar 7;9(3):147. doi: 10.3390/genes9030147 (PMC5867868; doi:10.3390/genes9030147)
Supplement: Supplementary file 1 [file genes-09-00147-s001.pdf]

Additional file 1: Figure S1. The geographical distribution of *E. nutans* and *E. sibiricus* accessions used in the study

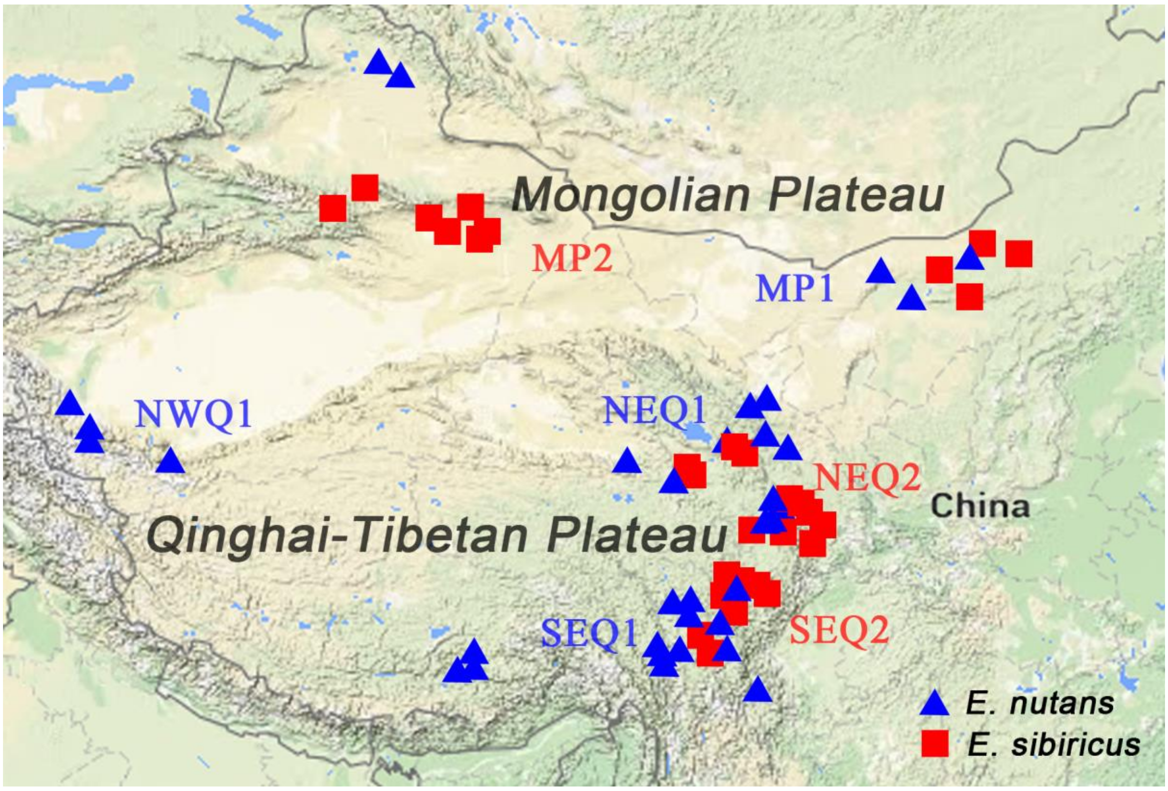

Additional file: Figure S2. The amplification results of 73 *Elymus* accesions by Elw2807s159

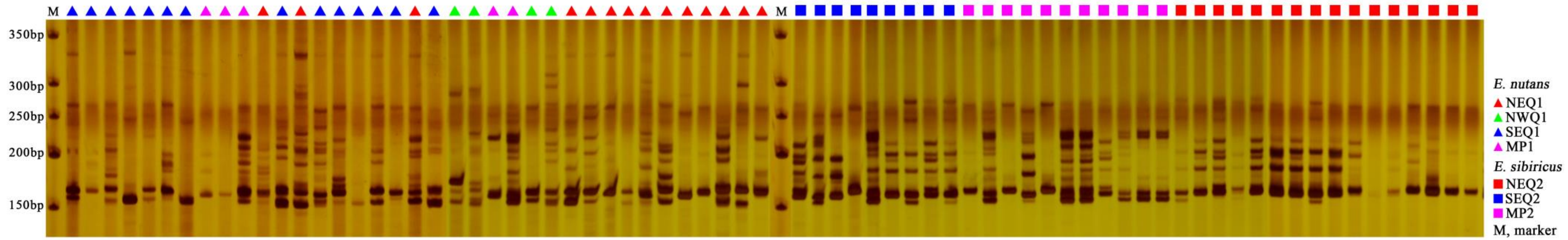

Additional file 2: Table S1. Details of seventy-three *Elymus* accessions used in the study

Group	Code	Accession No.	Origin	Status	Altitude
E. nutans					
NEQ1	1	PI639852	Xiahe, Gansu	wild material	2960m
NEQ1	2	PI655193	Maqu, Gansu	wild material	3280m
NEQ1	3	PI639855	Luqu, Gansu	wild material	3060m
NEQ1	4	Xiahe15	Xiahe, Gansu	wild material	3100m
NEQ1	5	PI628698	Tianzhu, Gansu	wild material	2860m
NEQ1	6	Xiahe48	Xiahe, Gansu	wild material	3100m
NEQ1	7	PI655192	Maqu, Gansu	wild material	3660m
NEQ1	8	PI655186	Xiahe, Gansu	wild material	2830m
NEQ1	9	PI499451	Lanzhou, Gansu	wild material	1520m
NEQ1	10	PI499611	Gulang, Gansu	wild material	2300m
NEQ1	11	PI531644	Gansu	wild material	2600m
NEQ1	12	PI499612	Xining, Qinghai	wild material	2450m
NEQ1	13	PI619586	Dulan, Qinghai	wild material	2950m
NEQ1	14	PI531645	Qinghai	wild material	2800m
NWQ1	15	PI619576	Qeshqer Shehiri, Xinjiang	wild material	2150m
NWQ1	16	PI628675	Hotan Prefecture, Xinjiang	wild material	3300m
NWQ1	17	PI619574	Qeshqer Shehiri, Xinjiang	wild material	3800m
NWQ1	18	PI619578	Qeshqer Shehiri, Xinjiang	wild material	2800m
SEQ1	19	PI619516	Sichuan	wild material	4100m
SEQ1	20	PI619569	Dege, Sichuan	wild material	4110m
SEQ1	21	PI619521	Luhuo, Sichuan	wild material	3740m
SEQ1	22	PI619520	Barkam, Sichuan	wild material	3300m
SEQ1	23	W6 22107	Kangding, Sichuan	wild material	3800m
SEQ1	24	W6 22112	Luhuo, Sichuan	wild material	3190m
SEQ1	25	PI531643	Omei, Sichuan	wild material	2900m
SEQ1	26	PI619522	Gongjue, Tibet	wild material	4100m
SEQ1	27	PI619590	Changdu, Tibet	wild material	3740m
SEQ1	28	PI619525	Tibet	wild material	3100m
SEQ1	29	PI619592	Changdu, Tibet	wild material	4200m
SEQ1	30	PI619527	Lhasa, Tibet	wild material	4460m
SEQ1	31	PI619530	Lhasa, Tibet	wild material	4020m
SEQ1	32	PI619532	Yangbajain, Tibet	wild material	4150m
MP1	33	PI547394	Inner Mongolia	wild material	1500m
MP1	34	PI499450	Inner Mongolia	cultivated material	1240m
MP1	35	PI504459	Inner Mongolia	cultivated material	1400m
MP1	36	PI619519	Burqin, Xinjiang	wild material	450m
MP1	37	PI619575	Habahe, Xinjiang	wild material	1200m
E. sibiricus					
NEQ2	38	XH02	Xiahe, Gansu	wild material	2510m

NEQ2	39	XH03	Xiahe, Gansu	wild material	2896m
NEQ2	40	XH09	Xiahe, Gansu	wild material	3171m
NEQ2	41	LQ04	Luqu, Gansu	wild material	3023m
NEQ2	42	LQ01	Luqu, Gansu	wild material	3010m
NEQ2	43	HZ01	Hezuo, Gansu	wild material	2715m
NEQ2	44	HZ02	Hezuo, Gansu	wild material	2900m
NEQ2	45	ZhN01	Zhuoni, Gansu	wild material	3218m
NEQ2	46	ZhN06	Zhuoni, Gansu	wild material	2678m
NEQ2	47	MQ01	Maqu, Gansu	wild material	3475m
NEQ2	48	LT01	Lintan, Gansu	wild material	2889m
NEQ2	49	LT02	Lintan, Gansu	wild material	3010m
NEQ2	50	Tongde	Qinghai	cultivar	3200m
NEQ2	51	Qingmu No.1	Qinghai	cultivar	3500m
NEQ2	52	PI531669	Xining, Qinghai	wild material	2400m
NEQ2	53	PI504462	Xining, Qinghai	wild material	2300m
SEQ2	54	Y1005	Ruoergai, Sichuan	wild material	3700m
SEQ2	55	SAU003	Kangding, Sichuan	wild material	3000m
SEQ2	56	SAU133	Aba, Sichuan	wild material	2900m
SEQ2	57	SAU139	Xinduqiao, Sichuan	wild material	3500m
SEQ2	58	SAU137	Lixian, Sichuan	wild material	2100m
SEQ2	59	SC02	Ruoergai, Sichuan	wild material	3500m
SEQ2	60	SC03	Ruoergai, Sichuan	wild material	3500m
SEQ2	61	Hongyuan	Hongyuan, Sichuan	breeding line	3500m
SEQ2	62	Chuancao No.2	Hongyuan, Sichuan	cultivar	3500m
MP2	63	PI499453	Xilinhot, Inner Mongolia	wild material	1000m
MP2	64	PI665507	Mongolia	wild material	1524m
MP2	65	PI499456	Shandan, Gansu	cultivated material	1900m
MP2	66	PI610886	Mongolia	wild material	920m
MP2	67	PI655140	Urumqi, Xinjiang	wild material	1650m
MP2	68	PI595182	Urumqi, Xinjiang	wild material	1250m
MP2	69	PI595180	Urumqi, Xinjiang	wild material	1600m
MP2	70	Y2003	Habahe, Xinjiang	wild material	500m
MP2	71	PI619577	Urumqi, Xinjiang	wild material	2300m
MP2	72	PI499462	Urumqi, Xinjiang	wild material	1050m
MP2	73	PI499468	Urumqi, Xinjiang	cultivated material	1770m

NEQ1, northeastern groups of *E. nutans* in Qinghai-Tibetan Plateau; NWQ1, northwestern groups of *E. nutans* in Qinghai-Tibetan Plateau; SEQ1, southeastern groups of *E. nutans* in Qinghai-Tibetan Plateau; MP1, Mongolian Plateau groups of *E. nutans*; NEQ2, northeastern groups of *E. sibiricus* in Qinghai-Tibetan Plateau; SEQ2, southeastern groups of *E. sibiricus* in Qinghai-Tibetan Plateau; MP2, Mongolian Plateau groups of *E. sibiricus*.

Additional file 3: Table S2. Correlation coefficients obtained from pairwise comparisons of 15 phenotypic traits of *E. nutans* (above diagonal) and *E. sibiricus* (below diagonal).

	**FL**	**FW**	**LB**	**WB**	**PH**	**CN**	**TN**	**CD**	**PL**	**FN**	**LL**	**WL**	**AL**	**SW1**	**SS**
FL(cm)		0.501**	0.915**	0.442**	-0.299	0.448**	0.409*	-0.038	0.322	0.524**	0.137	0.023	-0.010	0.197	-0.262
FW(cm)	0.695**		0.560**	0.941**	0.195	0.414*	0.195	0.280	0.331*	0.178	0.277	0.168	0.217	0.388*	-0.438**
LB(cm)	0.646**	0.702**		0.556**	-0.265	0.345*	0.349*	0.059	0.311	0.525**	-0.001	-0.036	-0.032	0.131	-0.306
WB(cm)	0.632**	0.846**	0.570**		0.246	0.355*	0.185	0.385*	0.399*	0.179	0.16	0.033	0.220	0.275	-0.509**
PH(cm)	0.110	0.228	0.288	0.176		0.202	-0.214	0.521**	0.262	-0.145	0.337*	0.21	0.379*	0.182	0.017
CN(No.)	-0.015	0.282	0.158	0.227	0.151		0.014	0.005	0.221	0.253	0.411*	0.217	0.231	0.401*	-0.054
TN(No.)	0.141	0.040	0.265	-0.137	0.143	0.091		-0.058	0.175	0.532**	-0.012	-0.147	0.002	-0.113	-0.058
CD(cm)	0.167	0.251	0.429**	0.237	0.603**	-0.173	0.168		0.232	-0.111	0.050	-0.073	0.112	0.003	-0.246
PL(cm)	-0.031	0.095	0.076	0.096	0.410*	-0.198	-0.082	0.410*		0.447**	0.073	0.083	0.014	0.114	-0.212
FN(No.)	-0.135	0.093	0.010	0.188	-0.034	0.011	-0.152	-0.074	0.246		-0.111	-0.114	0.023	-0.173	-0.062
LL(cm)	-0.006	0.103	-0.055	0.082	0.203	-0.303	0.075	0.404*	0.431**	0.19		0.576**	0.257	0.584**	0.009
WL(cm)	0.085	0.161	0.002	0.236	-0.119	0.255	-0.026	-0.310	-0.065	0.172	-0.125		-0.265	0.643**	0.192
AL(cm)	-0.148	0.180	0.134	0.082	-0.075	0.245	-0.086	-0.065	0.302	0.308	0.101	-0.008		-0.055	-0.096
SW1(g)	-0.137	-0.064	-0.177	0.086	0.128	-0.137	-0.053	0.293	0.428**	0.263	0.369*	0.343*	0.140		0.231
SS(gf)	-0.221	-0.053	-0.133	-0.017	0.187	0.009	-0.102	0.165	0.085	-0.026	0.244	0.257	0.261	0.409*	

** correlation is significant at the 0.01 level; * correlation is significant at the 0.05 level.

Additional file 4: Table S3. EST-SSR markers used in this study, amplification results, assigned linkage groups of *Elymus.*

**Primer**	***E. nutans***				***E. sibiricus***				**Assigned *Elymus* Chromosome**
	**T**	**TP**	**%P**	**PIC**	**T**	**TP**	**%P**	**PIC**	
Elw0300s019	10	10	100.00	0.33	8	8	100.00	0.22	1St
Elw0669s043	6	6	100.00	0.31	12	12	100.00	0.23	3H
Elw1197s069	7	7	100.00	0.41	7	6	85.71	0.30	6St
Elw1420s081	8	8	100.00	0.27	11	11	100.00	0.30	6H
Elw1468s087	11	11	100.00	0.38	11	11	100.00	0.33	6H
Elw1675s092	11	11	100.00	0.31	10	9	90.00	0.35	5H
Elw2676s146	16	15	93.75	0.38	10	9	90.00	0.12	2St
Elw2698s152	9	9	100.00	0.30	9	9	100.00	0.33	1H
Elw2807s159	11	11	100.00	0.42	11	11	100.00	0.43	6H
Elw2808s160	4	3	75.00	0.27	4	4	100.00	0.19	1St
Elw3264s184	9	9	100.00	0.39	6	6	100.00	0.37	7St
Elw3384s187	17	17	100.00	0.34	16	16	100.00	0.26	3H
Elw3492s190	12	12	100.00	0.35	9	7	77.78	0.19	7St
Elw3995s226	7	7	100.00	0.40	7	7	100.00	0.43	2H
Elw4419s261	8	7	87.50	0.26	8	7	87.50	0.12	5H
Elw5447s306	7	7	100.00	0.22	9	9	100.00	0.41	5St
Elw5616s393	23	23	100.00	0.16	22	22	100.00	0.38	1H
Elw5627s404	18	18	100.00	0.36	17	17	100.00	0.38	2H
Ps2283	14	14	100.00	0.36	13	13	100.00	0.28	4St
Ps261	8	7	87.50	0.29	9	9	100.00	0.34	4St
Ps3447	18	18	100.00	0.38	13	13	100.00	0.28	5St
Ps3577	6	6	100.00	0.29	5	5	100.00	0.26	5St
Ps938	13	13	100.00	0.28	13	12	92.31	0.29	7St
Ltc0209	13	13	100.00	0.35	12	11	91.67	0.30	
Ltc0096	9	9	100.00	0.34	8	6	75.00	0.20	
ES-7	4	3	75.00	0.32	4	1	25.00	0.12	
ES-22	11	11	100.00	0.35	7	2	28.57	0.03	

ES-23	18	18	100.00	0.31	14	12	85.71	0.12
ES-51	13	13	100.00	0.36	14	14	100.00	0.29
ES-97	8	7	87.50	0.30	8	4	50.00	0.10
ES-123	12	12	100.00	0.26	12	8	66.67	0.16
ES-125	5	4	80.00	0.30	5	2	40.00	0.15
ES-144	6	6	100.00	0.30	5	5	100.00	0.23
ES-231	7	6	85.71	0.20	6	5	83.33	0.06
ES-236	6	6	100.00	0.25	6	3	50.00	0.10
ES-253	12	11	91.67	0.27	12	8	66.67	0.21
ES-259	11	11	100.00	0.30	11	11	100.00	0.23
ES-310	10	10	100.00	0.39	9	3	33.33	0.04
ES-347	5	5	100.00	0.34	5	2	40.00	0.04
ES-405	2	2	100.00	0.45	2	1	50.00	0.23
Mean	10.1	9.9	96.59	0.32	9.5	8.3	82.73	0.23

T,total number of amplified bands; TP, total number of polymorphic bands; %P, percentage of polymorphism; PIC, polymorphic information content.

Predicted wheat/rice chromosomes and assigned *Elymus*/*Leymus* chromosomes are based on Larson and Kellogg (2009) and Mott et al. (2011)
